# Genome-Wide Identification and Expression Analysis of the SPL Gene Family in Three Orchids

**DOI:** 10.3390/ijms241210039

**Published:** 2023-06-12

**Authors:** Xuewei Zhao, Mengmeng Zhang, Xin He, Qinyao Zheng, Ye Huang, Yuanyuan Li, Sagheer Ahmad, Dingkun Liu, Siren Lan, Zhongjian Liu

**Affiliations:** 1 College of Forestry, Fujian Agriculture and Forestry University, Fuzhou 350002, China; 2 Key Laboratory of National Forestry and Grassland Administration for Orchid Conservation and Utilization, College of Landscape Architecture and Art, Fujian Agriculture and Forestry University, Fuzhou 350002, China

**Keywords:** SPL genes, Orchidaceae, phylogenetic analysis, flower development, expression analysis

## Abstract

*SPL* transcription factors regulate important processes such as plant growth and development, metabolic regulation, and abiotic stress. They play crucial roles in the development of flower organs. However, little is known about the characteristics and functions of the *SPL*s in the Orchidaceae. In this study, *Cymbidium goeringii* Rchb. f., *Dendrobium chrysotoxum* Lindl., and *Gastrodia elata* BI. were used as research objects. The SPL gene family of these orchids was analyzed on a genome-wide scale, and their physicochemical properties, phylogenetic relationships, gene structures, and expression patterns were studied. Transcriptome and qRT-PCR methods were combined to investigate the regulatory effect of *SPL*s on the development of flower organs during the flowering process (bud, initial bloom, and full bloom). This study identifies a total of 43 *SPL*s from *C. goeringii* (16), *D. chrysotoxum* (17), and *G. elata* (10) and divides them into eight subfamilies according to the phylogenetic tree. Most SPL proteins contained conserved SBP domains and complex gene structures; half of the genes had introns longer than 10 kb. The largest number and variety of cis-acting elements associated with light reactions were enriched, accounting for about 45% of the total (444/985); 13/43 *SPL*s contain response elements of *miRNA156*. GO enrichment analysis showed that the functions of most *SPL*s were mainly enriched in the development of plant flower organs and stems. In addition, expression patterns and qRT-PCR analysis suggested the involvement of *SPL* genes in the regulation of flower organ development in orchids. There was little change in the expression of the *CgoSPL* in *C. goeringii*, but *DchSPL9* and *GelSPL2* showed significant expression during the flowering process of *D. chrysotoxum* and *G. elata*, respectively. In summary, this paper provides a reference for exploring the regulation of the SPL gene family in orchids.

## 1. Introduction

Transcription factors (TFs) regulate the expression of target genes in plant cells by combining their DNA binding domains with *cis*-acting elements in the upstream promoter region of target genes and play an important role in plant morphogenesis and abiotic stress regulation [1,2]. *SQUAMOSA promoter-binding protein-like* (*SPL*) transcription factors were first identified in *Antirrhinum majus* L., named *SBP1* and *SBP2* according to their ability to bind *SQUAMOSA* genes, and these two transcription factors were involved in the early flower development of *A. majus* [3]. The SPL gene has a highly conserved SBP domain (composed of 76 amino acid residues), which contains two specific zinc finger motifs, Zn-1 (Cys-Cys-Cys-His) and Zn-2 (Cys-Cys-His-Cys), and a nuclear localization signal (NLS) at the C-terminal [4]. SPL transcription factors act during multiple stages of plant growth and development. They play an important role in the initiation of flowering [5,6], the development of stems and leaves [7,8], the formation of flowers and fruits [9,10], the transition from the vegetative stage to the reproductive stage [11,12], the response to abiotic stress [13,14,15], and the transduction of plant hormones (e.g., gibberellic acid) [16].

Research shows that most members of the SPL gene family contain the corresponding element (MRE) of *miRNA156*, which is generally located in the last exon of its domain [17,18]. Among the 17 *SPL*s in *Arabidopsis thaliana* (L.) Heynh., 11 are *miRNA156* target genes [19]. According to the size of the SBP domain, it can be divided into two types. One is *AtSPL9/15*, which mainly regulates the formation of leaves and the initiation of flowering. The other is *AtSPL3/4/5*, which mainly regulates flower morphology [6,20,21]. An increase in *miRNA156* expression inhibits the expression of these *SPL*s. At the same time, SPL also affects the downstream *miRNA172* and MADS-box genes to regulate the transformation of plant growth stages, the development of flowers and leaves, the synthesis of secondary metabolites, and the stress of high temperature, drought, and salt [22,23].

Orchidaceae is one of the largest angiosperm families in the world, with rich flower morphology, special pollination mechanisms, unique drought physiology, and very complex mycorrhizal relationships. These characteristics exhibit more unique specialization than any other plant on earth [24,25,26,27]. Orchids have three life forms: terrestrial, epiphytic, and saprophytic, making them an ideal group for studying biodiversity and evolution [28]. The flowers of orchids are usually symmetrical on both sides, including the sepals, petals, lip, and gynostemium, with the lip having the most complex morphological changes [29,30]. *Cymbidium goeringii* Rchb. f., *Dendrobium chrysotoxum* Lindl., and *Gastrodia elata* BI. are three different life forms of orchids with important ornamental and medicinal values [25,31,32,33]. The regulatory role of the SPL gene family in the growth process of many plants has been gradually revealed, but relatively little research has been done on orchids. *MiRNA156*/*SPL* synergistically regulates the development of reproductive organs and the perianth in *C. goeringii* [17]. The complementary expression patterns of *miRNA156* and its target gene *SPL* may mediate the transition from the nutritional to reproductive growth stages in *Phalaenopsis aphrodite* Rchb. f. [34]. In addition, *DcSPL3* was shown to be associated with plant maturation in *Dendrobium catenatum* Lindl [35]. In recent years, sequencing of the orchid genome has provided strong data support for studies on the origin, diversity, and functional gene identification of orchid species, making it possible to investigate more deeply the functions of the SPL gene family in orchid flower organ development [28,36].

In this study, gene structure analysis, phylogenetic tree construction, collinearity analysis, *cis*-acting element analysis, and expression pattern analysis of the SPL gene family in three orchid species were carried out to elucidate the characteristics of the *SPL*s during orchid floral development. Exploring the regulation function of the SPL gene family in the flower organ development process of different life forms of orchids is of great significance for the breeding and development of orchid resources. This result can provide new insights into the molecular mechanisms of floral organ development and morphological diversity in orchids.

## 2. Results

### 2.1. Identification and Physicochemical Properties

Through the screening of Blast and HMMER, 43 genes with complete SPL domains were finally obtained from three orchid species (16 in *C. goeringii*, 17 in *D. chrysotoxum,* and ten in *G. elata*). The *SPL*s were named according to the sequence of gene distribution on the chromosome (from top to bottom) (Table 1). The physicochemical properties of these SPL proteins vary greatly. The amino acid (aa) ranges from 219 to 1166 aa, and the molecular weight (Mw) ranges from 24.48 to 128.59 kDa. Moreover, half of the 43 *SPL*s are basic proteins (isoelectric point (pI) higher than 8.00), and the other half are neutral or weakly acidic (isoelectric point range from 5.37 to 7.99). In addition, the grand average of hydropathicity (GRAVY) of 43 SPL proteins is predicted to be negative, indicating that they are all hydrophilic proteins. The instability index (II) of the SPL members of the three orchids all exceeded 40, suggesting they were unstable proteins. The results of subcellular localization prediction show that most SPL proteins are located in the nucleus and a few are located in the cytoplasm, which indicates that SPL proteins play a role in the nucleus like most transcription factors, and some members also perform in the cytoplasm.

### 2.2. Phylogenetic Analysis

**Figure 1:**
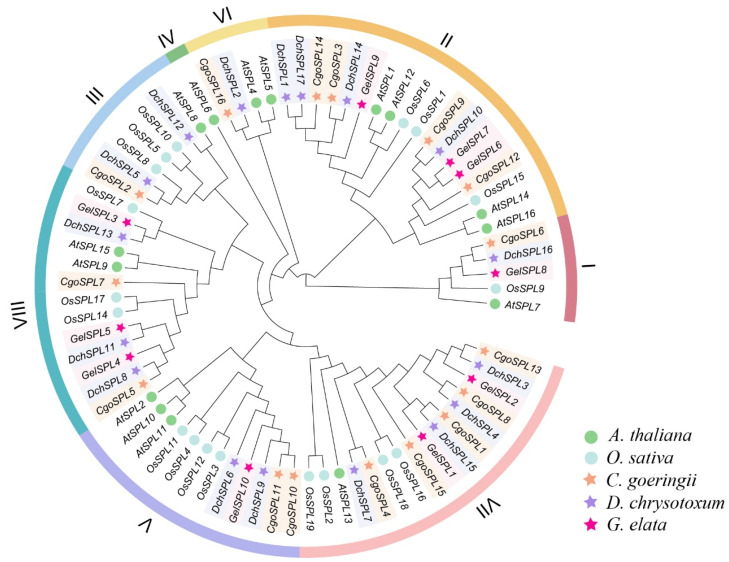
Phylogenetic tree of *SPL*s in five plants. The SPL gene family is divided into eight subfamilies (I–VIII), and the IV subfamily doesn’t contain the *SPL*s of orchids. The SPL protein sequence of orchids can be obtained in Table S1.

In order to further understand the evolutionary relationship between the SPL gene members of three orchids, a phylogenetic tree was constructed from the SPL protein sequence (76 members in total) of *C. goeringii* (*CgoSPL*), *D. chrysotoxum* (*DchSPL*), *G. elata* (*GelSPL*), *A. thaliana* (*AthSPL*), and *Oryza sativa* L. (*OsSPL*), which was divided into eight subfamilies (Figure 1). The phylogenetic tree showed that the *SPL*s of orchids had a closer relationship with *O. sativa*. Among the eight identified subfamilies, the IV subfamilies contained only one member of *A. thaliana* (*AthSPL6*), and the other subfamilies contained different numbers of *SPL*s of orchids. Subfamilies II and VII contained the largest number of *SPL*s in orchids, with 11 members. In addition, it was interesting that *CgoSPL*s and *DchSPL*s were included in subfamilies VI and III but not *GelSPL*s, which might be caused by the contraction of the SPL gene family of *G. elata*.

### 2.3. Protein Conservative Domain and Gene Structure Analysis

**Figure 2:**
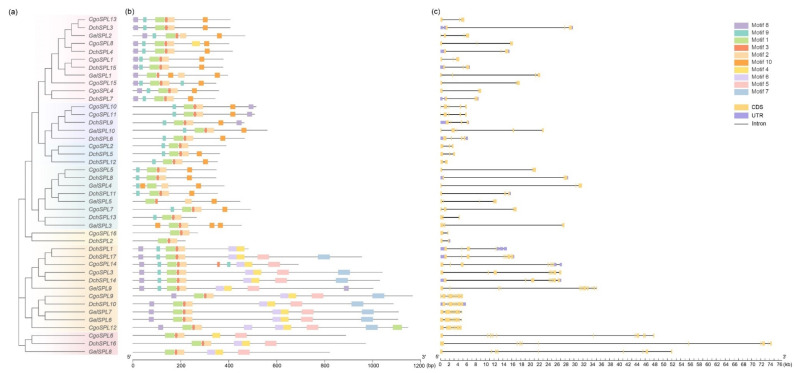
Phylogenetic relationships, motif, and structure of *SPL*s in orchids. (**a**) MEGA7.0 was used to construct a phylogenetic tree of 43 *SPL*s. (**b**) Use the conserved motif of the predicted SPL proteins on MEME. (**c**) Visualize the structure of SPL genes based on gff. The motif 1–10 sequence and logo are in Table S2.

**Figure 3:**
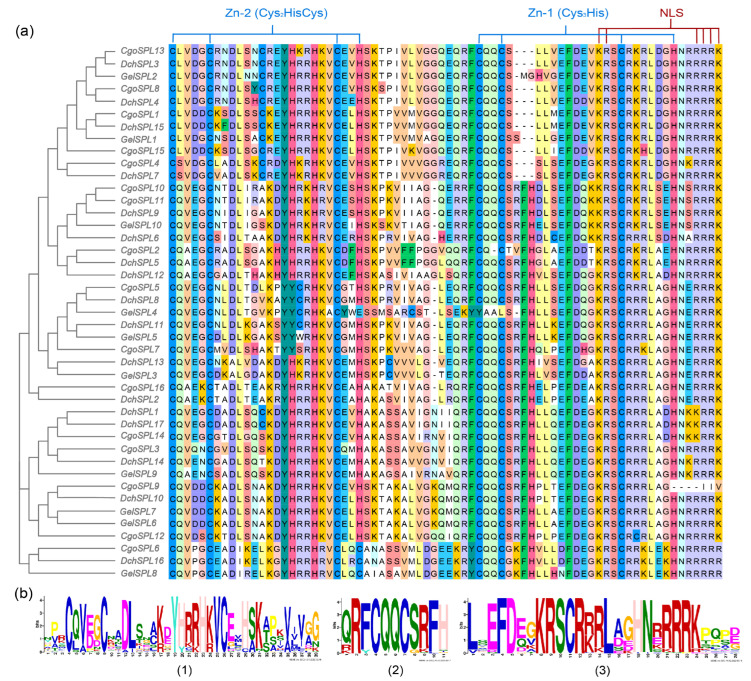
Conserved motifs in the SPL protein amino acid sequences. (**a**) SPL protein sequence alignment results. (**b**) Sequence logos of the Zn-1, Zn-2, and NLS domains.

Predicting the conserved domains of proteins can provide a reference for further study of protein functions. The online prediction website MEME analyzes the conservative domains of 43 SPL proteins, which are set as Motif1–Motif10 (Figure 2b). The results show that the distribution of the conserved domains of proteins with the same branches is similar. Each subfamily has different degrees of motif deletion, but all members contain motif 1, motif 2, and motif 3, which is the most conservative region in SPL proteins (Figure 3). The two proteins of the VI subfamily only have the most conservative regions and do not contain other conservative domains. Interestingly, motif 4, motif 5, and motif 6 almost only exist in subfamilies I and II. At the same time, these two subfamilies do not contain motif 10. In addition, motif 8 is near the C-terminal in the VI and II subfamilies but near the N-terminal in the V subfamily and does not exist in other subfamilies. Motif 7 has a large number of missing phenomena that only exist in some members of the II subfamily. The range and order of the motif provide the possibility for the protein to have more functions.

The intron-exon structure of the gene can be clearly seen by analyzing the SPLs of orchids through the online software GSDS. The 43 SPLs have different-length introns and members with close phylogenetic relationships and similar structures (Figure 2c). Among them, the intron length of the I subfamily genes is the longest, both exceeding 40 kb. The intron length of the VI subfamily genes is the shortest, less than 4 kb. An exciting phenomenon in subfamily II is that the phylogenetic tree divides 11 members into two branches. The gene structure in a single branch is very similar, but the gene structure between the two branches is different. In addition, CDS and UTR also have a variable distribution in these genes, and there are more CDS in genes with longer introns. The complex structure of genes makes their functions more variable.

### 2.4. Collinearity and Location Analysis on Chromosome

TBtools is used to visualize the distribution of 43 SPL genes on chromosomes. The results show that the SPLs of the three orchids are distributed on different chromosomes (Figure S1). The 16 *CgoSPL*s are distributed on 13 chromosomes, of which Chr10 has three *CgoSPL*s. The 17 *DchSPL*s are evenly distributed on 11 chromosomes; *DchSPL17* is distributed on unknown chromosomes; and 1–2 SPLs are placed on each chromosome. The ten *GelSPLs* are distributed on seven chromosomes, of which Chr08 has three *GelSPLs*, Chr03 has two *GelSPLs*, and the other chromosomes have only one.

**Figure 4:**
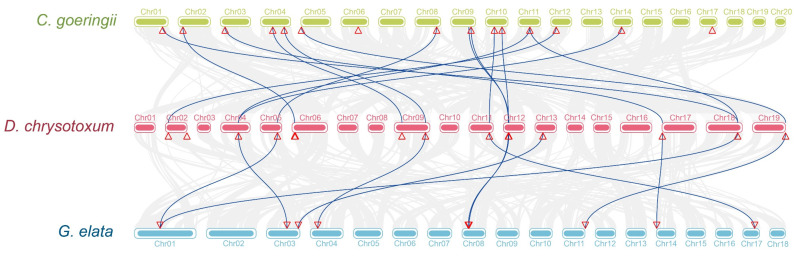
Collinearity analysis of *SPL*s of three orchids. The location of the *SPL*s is marked by a red triangle, and the blue lines show *SPL*s with collinear relationships between different species.

In addition, the collinear relationship between the SPLs of *C. goeringii*, *D. chrysotoxum*, and *G. elata* is analyzed to identify potential replication events in the evolution of the SPL genes in orchids. The results show that most of the SPL genes in the three orchids have a collinear relationship. Although the SPLs of *G. elata* contract significantly, they still correspond to the SPLs of the other orchids (Figure 4). This indicates that the SPL genes of orchids have a high degree of homology. In addition, we combine gene locations on chromosomes to find potential repetitive events. This indicates that the SPL genes of the three orchids underwent less direct homologous recombination and more genomic rearrangements during their evolution.

### 2.5. Cis-Elements Analysis

**Figure 5:**
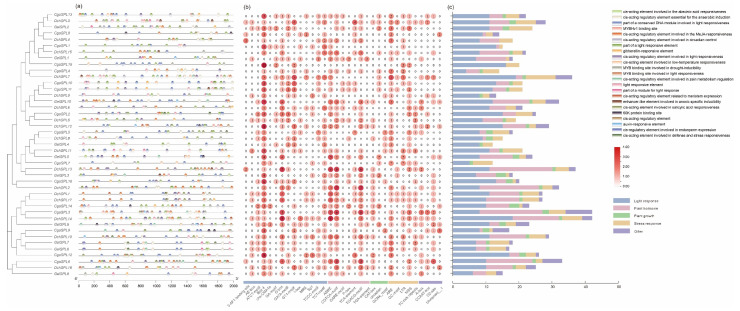
The *cis*-acting element in the *SPL* promoter region. (**a**) The distribution of *cis*-acting elements at 2000 bp upstream of the *SPL*s; (**b**) The number of *cis*-acting elements in the promoter region; (**c**) Count the number of light response, plant hormone, plant growth, and stress response elements for each SPL gene. The captions are marked on the right, and the types and quantities of *cis*-acting elements are shown in Table S3.

We searched the *cis*-elements present in the promoter region of 2000 bp upstream of 43 genes to further study the regulatory function of SPLs in orchids. Through research, we have obtained a total of 33 *cis*-elements, for a total of 985 *cis*-elements (Table S3). Changes in the type and quantity of elements provide more possibilities for gene function. In this study, *DchSPL14* and *CgoSPL3* contain 42 *cis*-elements, which is the largest number (Figure 5a). Box 4 is a relatively common photoreaction-related element with the largest number (135/985) (Figure 5b). In addition, among the identified elements, the types and quantities of photoreactively related elements are the highest, with a total of 14 types accounting for approximately 45% (444/985) of the total. There are seven *cis*-elements related to plant hormone regulation and five *cis*-elements related to stress, accounting for approximately 26% (258/985) and 13% (159/985), respectively. The number of elements related to plant growth regulation is the lowest, with only three species accounting for approximately 5% (49/985). The SPL genes of *G. elata* contain significantly fewer photoreactive *cis*-elements than those of the other two orchids (Figure 5c).

### 2.6. GO Analysis

**Figure 6:**
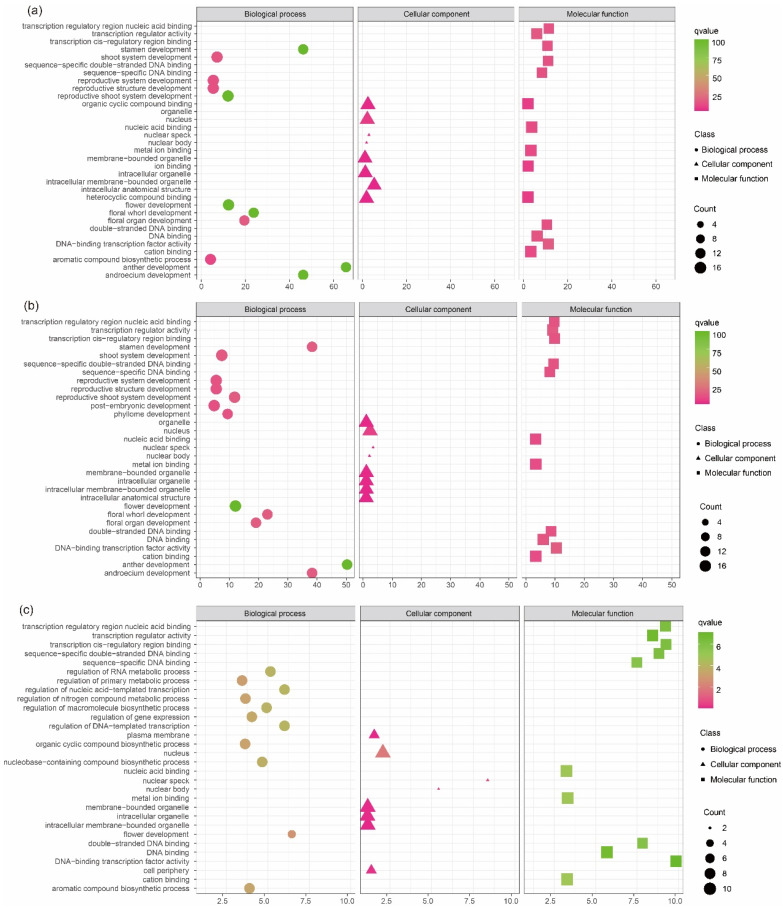
Gene ontology (GO) terms of *SPL*s of *C. goeringii* (**a**), *D. chrysotoxum* (**b**), and *G. elata* (**c**).

We attempt to use GO to more comprehensively describe the functions and properties of SPL proteins in orchids, revealing that they may participate in a series of biological processes (BP), cellular components (CC), and molecular functions (MF). The results show that among the three different life forms of orchids, the enrichment status of *C. goeringii* and *D. chrysotoxum* is relatively similar, while the accumulation of SPL protein in *G. elata* is less significant, especially in BP and MF. The proportion of genes enriched in BP is relatively large, but some proteins are not significantly enriched. Its function is mainly related to the development of the floral organ, stamens, and shoot in plants. Like most plants, the membership in CC is relatively low, but its enrichment is significant and mainly related to organelles. Most of the proteins enriched in MF have the ability to interact with other proteins, especially the *CgoSPL* proteins (Figure 6).

### 2.7. Expression Pattern Analysis of SPLs in Orchids

**Figure 7:**
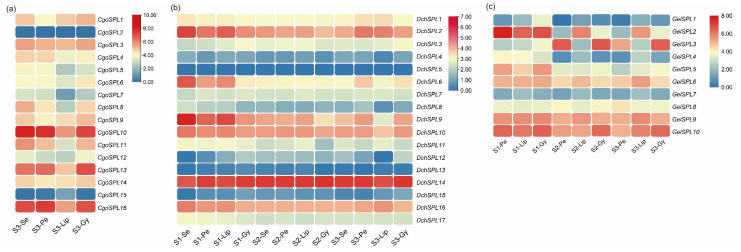
Expression pattern of *SPL*s in the floral component of orchid. (**a**) Expression heatmap of SPL genes in *C. goeringii* at stage of full bloom. (**b**) Expression pattern of *SPL*s in flower components (sepals, petals, lip, gynostemium) of *D. chrysotoxum* at three flower development stages (bud, early flowering, full bloom). (**c**) Expression pattern of SPL genes in flower components (perianth tube, lip, and gynostemium) of *G. elata* at three flower development stages (bud, early flowering, and full bloom). The FPKM values of *SPL*s are in Table S4.

The expression spectrum shows that the expression of SPLs of the same subfamily of the same species is similar. For example, the gene expression of the subfamily III is lower in *C. goeringii* and *D. chrysotoxum*, and the expression of the subfamily VI is higher, but the SPLs of both subfamilies are missing in *G. elata*. However, there are also cases of species expression specificity in the same subfamily, such as drumsticks with high expression in subfamily II but lower expression in the other two. In addition, some genes also have tissue- or period-specific expression, and *CgoSPL6* and *GelSPL8* are expressed in the GY of *C. goeringii* and *G. elata* but lower in the GY of drumsticks. The subfamily V shows high expression in SE and GY during the development of spring orchids, but higher expression in pe and lip in *D. chrysotoxum* and *G. elata*. *CgoSPL*13 and *GelSPL*2 of subfamily VII show significant expression at S1, and the rest of the genes are barely expressed. The subfamily VIII exhibits low expression in *C. goeringii* and *D. chrysotoxum*, but the expression of *GelSPL*3 in PE and LIP is higher than in GY, and the expression of *GelSPL*5 in S1 is higher than its expression in other periods. This fluctuation may be related to the development of PE and LIP and flowering regulation (Figure 7).

### 2.8. qRT-PCR Analysis

**Figure 8:**
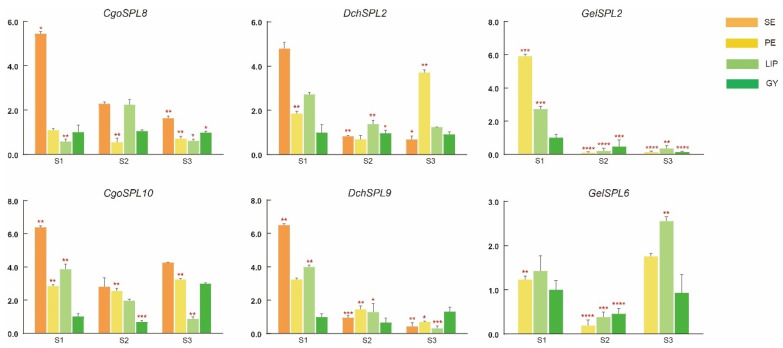
Real-time reverse transcription quantitative PCR (RT-qPCR) verifies the effect of *SPL*s on flower organ development. Y-axis represents relative expression values (2^−ΔΔCT^). Bars represent the mean values of three technical replicates ± SE. The red asterisk indicates the P value in the significance test (* *p* < 0.05, ** *p* < 0.01, *** *p* < 0.001, **** *p* < 0.0001). Primers and RT-qPCR analyses of SPL genes are shown in Table S5.

We selected candidate genes with different expressions in each species for qRT-PCR experiments based on transcriptome data (the sequence information of primers is in Table S1. The results show a significant correlation between the expression trend of the selected genes and the expression level of the transcriptome data (Figure 8). In *C. goeringii*, the expression of *CgoSPL8* and *CgoSPL10* is higher in sepals, especially in SE1. The expression of *CgoSPL8* is lower in petals, but the expression of *CgoSPL10* is significantly upregulated. The expression of *CgoSPL10* in the lip shows a downward trend with flowering. The expression of *DchSPL2* and *DchSPL9* is higher in *D. chrysotoxum* S1, and the expression is downregulated in both S2 and S3. However, interestingly, *DchSPL2* is significantly upregulated in S3-PE, which may be related to the development of *D. chrysotoxum* petals. In *G. elata*, *GelSPL2* shows an extremely significant decrease in both S2 and S3 periods but shows a significant upregulation in various parts of S1. *GelSPL6* expression is more stable than *GelSPL2* but significantly declines during S2.

## 3. Discussion

Since the establishment of Mendel’s laws, flowers have played an important role in the study of plant developmental biology. Orchids are one of the largest families of flowering plants, and the contraction and amplification of genes related to flower morphology can enhance our understanding of functional genes and gene evolution related to orchid flower development [39]. At present, research on the flower organs of orchids mainly focuses on the flowering process, floral morphology, color, and scent [28]. The SPL transcription factor is one of the key target genes of *miRNA156*, which is closely related to important processes such as plant growth and development, metabolic regulation, and abiotic stress and plays an important role in regulating the development of flower organs [5,40]. However, the characteristics and functions of the SPL gene have been poorly studied in orchids. Therefore, we used various methods to identify the SPL gene family of orchids and examined the evolution of SPL proteins and their functional properties. We identified 16 *CgoSPL*s, 17 *DchSPL*s, and 10 *GelSPL*s (Table 1), which were similar to the number of *SPL*s in other plants, such as *A. thaliana* [41] (17), *O. sativa* [42] (19)*, Betula luminifera* H. Winkl. [43] (18), and *Passiflora edulis* Sims. [44] (14). However, some studies have found significant expansion of the SPL gene in some dicots, such as *Fraxinus mandshurica* Rupr. [45] (36), *Glycine max* (Linn.) Merr. [46] (46), and *Brassica napus* L. [47] (58). Gene duplications resulting from genome-wide replication events or small-scale replication events in angiosperms are thought to play an important role in adaptation and generating evolutionary novelty [48,49]. Compared to the other two orchids, the SPL gene family of *G. elata* showed contractions, which might be related to the natural selection of mycoheterotrophy in orchids. These differences may be due to gene duplication events or the different frequencies of retained copies after duplication events.

Through the phylogenetic tree, the evolutionary relationships of species and the kinship of genes can be visually observed. The phylogenetic tree constructed a total of 76 *SPL*s and divided them into eight subfamilies (Figure 1). We show that the SPL gene family in orchids is more closely related to *O. sativa* than *A. thaliana*. *CgoSPL* and *DchSPL* are distributed in almost every subfamily and are more closely related and similar in number. However, *SPL*s from *O. sativa* and orchids are not included in IV, and whether these genes were lost during the evolution of monocotyledons remains to be explored. *GelSPL* is not included in subfamilies VI and III, and this missing gene may be the result of evolutionary selection in *G. elata*. In addition, the physical and chemical properties of 43 *SPL*s show abundant variations within the gene family. Gene replication events (segmental and tandem) are a major driver for the discovery of new genes and gene family expansion, supporting organisms’ adaptation to different complex environments [50,51]. In this study, the evolutionary relationship between the *SPL*s of three orchids is revealed through collinear analysis (Figure 4). The results show that the ratio of most *CgoSPL* and *DchSPL* genes is close to 1:1, indicating that there are no repeat events in *SPL*s after the differentiation of the two orchids. Although the number of *GelSPL*s is small, there is a one-to-one correspondence between the three genes. In addition, although there are fewer genes in *G. elata*, there is almost a one-to-one correspondence with the chromosomes of *C. goeringii* and *D. chrysotoxum*, indicating that there is no obvious change in the structure between chromosomes after the differentiation of the two species.

The structure of genes may also affect the phylogenetic relationship. During evolution, introns are considered to be one of the important reasons for the formation of new genes. Orchids have the characteristics of high heterozygosity and long introns [52,53]. In this study, by visualizing its gene structure and conserved motif order, it was found that the intron length of *SPL*s in orchids is longer than that of other species, and most *SPL*s have similar genetic structure and conserved motif order in the same subfamily. However, there are also differences between different branches of the same subfamily (Figure 2). The introns of subfamily I are all longer (more than 40 kb), and the introns of subfamilies III and VI are shorter (less than 5 kb). The intron lengths of the two branches in subfamily II differ greatly, but the number of CDS is similar, and Motif5 and Motif7 only appear in this subfamily.

*Cis*-elements participate in the dynamic network of gene regulation, thereby regulating the response of plants to the external environment. In this study, a large number of *cis*-elements related to light, hormones, and abiotic stress were found in the upstream 2000 bp promoter region of 43 *SPL*s, suggesting the diverse functions of this gene family in orchids (Figure 5). The miRNA is mainly regulated after transcription by cleaving the target gene’s RNA, and miRNA156 is currently the only known gene that can regulate plant age. Most of the identified *SPL*s in various non-model plants are target genes for *miRNA156*, such as *Zea mays* L. (19/31), *Petunia axillaris* (Lam.) Britton (14/21), and *Carica papaya* L. (7/14) [54,55,56]. However, among the identified *SPL*s in this trial, only 13 contain the response element (MRE) of *miRNA156*, and only *GelSPL9* in *G. elata* contains the MRE element. Our transcriptome data shows that the target genes of these *miRNA156* are expressed differently in different parts of the flowering process. In addition, most of the *SPL*s are enriched in flower development-related pathways in GO analysis, and these results show that *SPL*s are fully involved in the flower development process of orchids (Figure 8).

The expression pattern of a gene can directly affect its regulatory function. There are few reports on the function of the SPL gene in orchids, but transcriptome-level studies have been found in a variety of plants. Most *SPL*s of species such as *A. thaliana*, *O. sativa*, and *Populus trichocarpa* Torr. and Gray are expressed in their tissues, such as flowers, floral parts, juvenile spikelets, roots, stems, and leaves [57,58,59]. The expression of *BpSPL*s in apical buds and male inflorescences of *Betula platyphylla* is inversely proportional to the expression of *miRNA156*, suggesting that *BpSPL*s may be involved in flower development [14,60]. Overexpression of *FmSPL2* in transgenic *Nicotiana tabacum* L. resulted in taller plants, changes in the morphology and number of roots, rounded leaves, and an earlier flowering time [45]. In subfamily VII, *VcSPL20* was significantly down-regulated during floral organ development in *Vaccinium corymbosum* L., while *VcSPL35* was maintained at a high level [61]. *GelSPL2* and *CgoSPL8* in subfamily VII were significantly up-regulated in S1, but the expression of *DchSPL*s in this branch didn’t change significantly, and this subclade may be involved in the floral organ development of *C. goeringii* Rchb. f. and *G. elata*. On the basis of the transcriptome data, in order to further analyze the function of the SPL gene in the floral component of orchids, we selected six *SPL*s with different expressions in different species based on transcriptome data for qRT-PCR experiments. The experimental results are similar to the expression trend of the transcriptome, and the expression of these genes is significantly upregulated in the sepal/tepal of the S1 stage of three orchids. *CgoSPL8* is significantly upregulated in S1-SE, and *CgoSPL10* expression is upregulated in both SE and PE in three periods. Therefore, these two genes may be involved in the development of sepals and petals in *C. goeringii*. (Figure 7). *AthSPL3*/*4* is mainly involved in promoting the process of flowering [21]. In this study, *GelSPL2* shares the same branch with *AthSPL3*/*4*, suggesting a role in the regulation of *G. elata* flowering. The result of qRT-PCR shows that with the occurrence of the flowering process, the expression of the SPL gene in GY does not change significantly, but the expression of *DchSPL9* and *GelSPL2* is down-regulated. Together, the present findings confirm that the SPL gene family in Orchidaceae may regulate the development of sepals/tepals and promote the flowering processes of *C. goeringii*, *D. chrysotoxum*, and *G. elata*.

## 4. Materials and Methods

### 4.1. Plant Materials

The plant material selected for this study was obtained from wild-type plants grown under natural conditions in the greenhouse of the Forest Orchid Garden of Fujian Agriculture and Forestry University. Samples of flower parts (sepal, SE; petal, PE; lip, LIP; and gynostemium, GY) of three flower development stages (bud, S1; initial bloom, S2; full bloom, S3) of *C. goeringii*, *D. chrysotoxum*, and *G. elata* (sequence in Figure S2) were collected in liquid nitrogen and then stored in −80 °C refrigerator.

### 4.2. Identification and Physicochemical Properties of the SPLs

Using 17 *AthSPL*s (SPL genes of *A. thaliana*) as probes, blast identification was carried out in genome files of three orchids, respectively (TBtools v1.120, Blast Compare Two Seqs; E-value, 1 × 10^−5^) [62,63], and the possible sequences obtained were blasted again (Blastp, https://blast.ncbi.nlm.nih.gov/Blast.cgi?PROGRAM=blastp&PAGE_TYPE=BlastSearch&LINK_LOC=blasthome, accessed on 20 November 2022) in NCBI. At the same time, the conserved domains of SPL, PF03110, were downloaded from the online database [3] (http://pfam.xfam.org/, accessed on 20 November 2022) to perform the HMMER search (default parameters). Based on the results of Blast and HMMER, the genes obtained were analyzed with the NCBI Batch CDD (https://www.ncbi.nlm.nih.gov/Structure/bwrpsb/bwrpsb.cgi, accessed on 21 November 2022) to screen and retain the genes with complete SPL domains [63]. The online analysis software ExPASy (https://www.expasy.org/, accessed on 22 November 2022) was used to analyze the physical and chemical properties of the obtained proteins, such as the protein length, isoelectric point (pI), molecular weight (MW), hydrophilic large average (GRAVY), instability index (II), and fat index (AI) of the protein [64]. Snapgene was used to analyze the CDS length. The online tool Cell-PLoc 2.0 (http://www.csbio.sjtu.edu.cn/bioinf/Cell-PLoc-2/, accessed on 23 November 2022) was used to predict subcellular localization.

### 4.3. Phylogenetic Analysis

The sequences of 16 SPL proteins of *C. goeringii* (*CgoSPL*), 17 SPL proteins of *D. chrysotoxum* (*DchSPL*)*,* 10 SPL proteins of *G. elata* (*GelSPL*)*,* 17 SPL proteins of *A. thaliana* (*AthSPL*), and 19 SPL proteins of *O. sativa* (*OsSPL*) were introduced into MEGA 7.0 [10,65]. In detail, the alignment sequences selected with the ClustalW program, Gap Opening and Gap Extend, are 15 and 6.66, respectively; the DNA Weight Matrix selection is the IUB; other values keep the default. Then, the phylogeny test was performed using 1000 replications of the bootstrap method [66]. The online software Evloview (http://www.evolgenius.info/evolview/#/treeview, accessed on 27 November 2022) was used to improve and beautify the phylogenetic tree [67].

### 4.4. Protein Conservative Domain and Gene Structure Analysis

The online software MEME (https://meme-suite.org/meme/doc/meme.html, accessed on 30 November 2022) was used to analyze the conserved motifs of SPL proteins from three orchids, and the prediction number was set to ten [68]. Based on the gff file, the gene structure was analyzed using the online tool GSDS (http://gsds.gao-lab.org/, accessed on 10 December 2022) [69]. TBtools v1.120 was used to integrate phylogenetic trees, conserved protein motifs, and general comparative maps of gene structures.

### 4.5. Collinearity and Location Analysis on Chromosome

TBtools v1.120 was used to extract the location information of SPL genes from *C. goeringii*, *D. chrysotoxum*, and *G. elata* genome files and gene annotation files to construct the physical map of *SPL*s of three orchids on chromosomes. The One-Step MCscanX command in TBtools v1.120 was used to analyze the collinear relationship among the three species and to identify the collinear blocks of SPL genes in *C. goeringii*, *D. chrysotoxum*, and *G. elata* genome files.

### 4.6. Cis-Acting Regulatory Elements Analysis

The upstream 2000 base pair sequence of the promoter codon was obtained from the genomes of *C. goeringii*, *D. chrysotoxum*, and *G. elata* by using TBtools. The online software PlantCARE [70] (http://bioinformatics.psb.ugent.be/webtools/plantcare/html/, accessed on 29 December 2022) was used to analyze the *cis*-acting regulatory elements in the promoter region of the *CgoSPL*s, *DchSPL*s, and *GelSPL*s genes. Excel software was used to process data, and TBtools v1.120 and GraphPad Prism 9.0.0 software were used for visualization.

### 4.7. Gene Ontology Analysis

Gene ontology (GO) is an internationally standardized gene function classification system that conducts GO function enrichment analysis on differential genes to identify the functions of differential gene enrichment. This study is based on the Uniprot (Universal Protein) [71] database and uses the Go Seq R language pack to conduct GO enrichment analysis on members of the SPL gene family in orchids, revealing that they may participate in a series of cellular components, molecular functions, and biological processes.

### 4.8. Expression Pattern and qRT-PCR Analysis

In order to study the expression pattern of the *SPL*s in orchid floral development, RNA-Seq by Expectation Maximization (RSEM) [72] was used for transcription quantification, the calculation of the fragments per kilobase per million mapped reads (FPKM) of each gene was calculated, and the RNA sequencing transcriptome database of flower parts at different stages was established (three replicates were set for each sample). Then, the heat map was drawn in TBtools according to the FPKM.

Quantitative real-time PCR (qRT-PCR) was used to further analyze the expression pattern of the *SPL*s. Total RNA was extracted using a FastPure Plant Total RNA Isolation Kit (for polysaccharide- and polyphenol-rich tissues) (Vazyme Biotech Co., Ltd., Nanjing, China) from the flower parts of *C. goeringii* Rchb. f., *D. chrysotoxum*, and *G. elata* during three periods of flowering. First-strand DNA was synthesized with TransScript^®^ All-in-One First-Strand cDNA Synthesis SuperMix for quantitative PCR (TransGen Biotech, Beijing, China). Primer Premier 5 software was used to design primers for candidate genes and internal reference genes for qRT-PCR. A primer blast on the NCBI website was used to confirm primer specificity. Hieff^®^ qPCR SYBR Green Master Mix (Low Rox Plus) (Yeasen Biotechnology (Shanghai) Co. Ltd., Shanghai, China) was used for qRT-PCR assays. The genes *CgoActin*, *DchActin*, and *GelActin* were used as the reference genes (the sequences are shown in Table S1). Finally, we calculated the relative expression of the target genes by the 2^−△△CT^ method (using GY1 as a reference). The expression data were the mean of the three biological replicates.

## 5. Conclusions

In our study, 43 *SPL*s were identified for *C. goeringii* Rchb. f. (16)*, D. chrysotoxum* (17), and *G. elata* (10) in total and could be classified into eight subfamilies according to the phylogenetic relationship. Subfamilies II and VII accounted for the largest proportion amongst all subfamilies, with 11 members, respectively. The *SPL*s of orchids have integrally conserved domains, and genes from the same subfamily have similar genetic structures. Most of the *SPL*s have photosensitization-related regulatory elements and functions related to flower organ development. In addition, expression profiling and qRT-PCR analysis indicated that *SPL*s may be involved in the regulation of floral organ development during the flowering process of orchids, especially *DchSPL9* and *GelSPL2*, which may have an important impact on the regulation of flowering in orchids. These results provide a reference for further understanding of the involvement of the *SPL*s in the regulation of the flowering process in orchids and their effects on the growth of flowering organs.

## Figures and Tables

**Table 1:** Characteristics of the SPL proteins from Orchidaceae.

Name ^1^	ID	AA ^2^ (aa)	Mw ^3^ (kDa)	pI ^4^	II ^5^	AI ^6^	Gravy ^7^	CDS ^8^ (bp)	Chromosome Location ^9^	Subcellular Localization ^10^
*CgoSPL1*	*GL13531*	377	42.33	5.67	57.9	65.17	−0.602	1134	Chr01: 230031076-230035267	Nucleus.
*CgoSPL2*	*GL20345*	389	42.74	9.24	64.01	55.96	−0.547	1170	Chr02: 39176698-39179624	Nucleus.
*CgoSPL3*	*GL21357*	1040	115.28	6.96	60.46	79.24	−0.338	3123	Chr03: 37958000-37985156	Cytoplasm.
*CgoSPL4*	*GL00812*	358	39.90	8.34	53.58	58.04	−0.691	1077	Chr04: 98733351-98742543	Nucleus.
*CgoSPL5*	*GL20492*	348	37.73	8.85	42.31	57.79	−0.656	1047	Chr04: 192125302-192146773	Nucleus.
*CgoSPL6*	*GL13362*	888	100.57	6.77	45.24	87.64	−0.149	2667	Chr05: 4434453-4482550	Cytoplasm. Nucleus.
*CgoSPL7*	*GL09506*	490	53.11	9.57	57.68	56.39	−0.592	1473	Chr06: 141164766-141181893	Nucleus.
*CgoSPL8*	*GL18277*	401	44.00	6.24	56.56	62.72	−0.532	1203	Chr08: 176169836-176186187	Nucleus.
*CgoSPL9*	*GL21138*	1166	128.59	7.1	59.21	74.34	−0.447	3498	Chr09: 165067503-165072570	Cytoplasm. Nucleus.
*CgoSPL10*	*GL02243*	514	56.41	9.36	43.4	57.51	−0.645	1545	Chr10: 71076233-71082132	Cytoplasm. Nucleus.
*CgoSPL11*	*GL02624*	508	55.60	9.53	42.46	58.21	−0.632	1524	Chr10: 71249381-71255310	Nucleus.
*CgoSPL12*	*GL10271*	1147	125.51	8.16	53.34	78.49	−0.354	3444	Chr10: 133962266-133967116	Nucleus.
*CgoSPL13*	*GL18257*	406	44.52	6.01	57.76	65.54	−0.573	1221	Chr11: 95920477-95925824	Nucleus.
*CgoSPL14*	*GL13558*	690	76.76	7.93	48.54	81.22	−0.414	2073	Chr12: 43955282-43982564	Nucleus.
*CgoSPL15*	*GL10802*	347	38.81	6.32	57.82	69.42	−0.664	1044	Chr14: 71780070-71797878	Cytoplasm. Nucleus.
*CgoSPL16*	*GL08313*	270	30.07	9.5	75.19	61.93	−0.659	813	Chr17: 102450263-102452061	Nucleus.
*DchSPL1*	*Maker79039*	482	53.40	7.51	59.19	71.02	−0.602	1449	Chr02: 7843583-7858503	Nucleus.
*DchSPL2*	*Maker65322*	219	24.48	9.14	74.1	55.34	−0.787	660	Chr02: 58663177-58665354	Nucleus.
*DchSPL3*	*Maker61968*	406	45.04	6.02	59.57	67.22	−0.624	1221	Chr04: 48287280-48317053	Nucleus.
*DchSPL4*	*Maker96228*	416	46.29	6.83	52.45	74.98	−0.428	1251	Chr05: 46813915-46829419	Nucleus.
*DchSPL5*	*Maker109701*	362	39.95	9.08	64.74	54.65	−0.575	1089	Chr06: 6975945-6979210	Nucleus.
*DchSPL6*	*Maker112013*	466	51.85	8.52	48.11	63.45	−0.619	1401	Chr06: 9196495-9202663	Nucleus.
*DchSPL7*	*Maker68296*	343	38.01	6.75	59.03	59.97	−0.624	1032	Chr09: 20765814-20774403	Nucleus.
*DchSPL8*	*Maker74669*	347	37.67	8.97	51.51	60.2	−0.543	1044	Chr09: 85010872-85039578	Nucleus.
*DchSPL9*	*Maker57539*	464	50.63	7.67	44.54	59.48	−0.581	1395	Chr11: 55483197-55489630	Nucleus.
*DchSPL10*	*Maker56717*	1086	119.98	6.95	58.17	76.21	−0.438	3261	Chr12: 13543009-13548711	Nucleus.
*DchSPL11*	*Maker56713*	353	38.50	9.12	57.86	59.43	−0.582	1062	Chr12: 14936124-14951987	Nucleus.
*DchSPL12*	*Maker65199*	353	37.40	8.91	51.78	52.95	−0.512	1062	Chr13: 21531520-21533117	Nucleus.
*DchSPL13*	*Maker65105*	266	28.84	9.63	61.45	60.98	−0.607	801	Chr13: 21593186-21597503	Nucleus.
*DchSPL14*	*Maker58047*	1029	113.76	7.07	55.63	81.42	−0.348	3090	Chr17: 732323-759462	Nucleus.
*DchSPL15*	*Maker110154*	376	42.04	5.37	58.26	66.91	−0.557	1131	Chr18: 85766921-85773574	Nucleus.
*DchSPL16*	*Maker86855*	970	110.37	7.99	46.52	79.92	−0.371	2910	Chr19: 90135422-90210026	Cytoplasm. Nucleus.
*DchSPL17*	*Maker22024*	954	106.57	8.31	49.18	80.83	−0.345	2865	Unknow: 235138-251725	Cytoplasm.
*GelSPL1*	*Gel009276*	396	44.14	5.95	52.17	75.63	−0.231	1191	Chr01: 56565872-56588357	Nucleus.
*GelSPL2*	*Gel001761*	467	50.87	7.24	58.66	73.88	−0.453	1404	Chr03: 45183311-45189748	Cytoplasm. Nucleus.
*GelSPL3*	*Gel018509*	453	49.12	9.33	73.85	80.6	−0.084	1362	Chr03: 70218932-70246803	Nucleus.
*GelSPL4*	*Gel007541*	381	41.10	9.2	54.53	55.22	−0.492	1146	Chr04: 16387253-16419109	Nucleus.
*GelSPL5*	*Gel012145*	447	49.32	8.93	52.7	74.72	−0.22	1344	Chr08: 13481955-13494586	Nucleus.
*GelSPL6*	*Gel008706*	1105	121.28	7.48	58.57	76.99	−0.428	3318	Chr08: 15310685-15315556	Nucleus.
*GelSPL7*	*Gel013055*	1108	121.56	7.28	58.37	79.07	−0.396	3327	Chr08: 15375314-15380178	Nucleus.
*GelSPL8*	*Gel016438*	821	92.44	6.13	48.37	82.79	−0.263	2466	Chr11: 50235152-50287389	Nucleus.
*GelSPL9*	*Gel008682*	1002	112.49	8.4	54.79	87.12	−0.218	3006	Chr14: 375537-410747	Cytoplasm. Nucleus.
*GelSPL10*	*Gel015151*	560	60.91	8.27	46.54	59.43	−0.561	1683	Chr17: 25780944-25804212	Nucleus.

Note: ^1^ *SPL*s are named according to the position of genes on chromosomes; ^2^ amino acids; ^3^ molecular weights; ^4^ theoretical isoelectric points; ^5^ instability indexes; ^6^ aliphatic indexes; ^7^ grand averages of hydrophobicity; ^8^ Snapgene is used to calculate the CDS length of genes; ^9^ The location of the gene on the chromosome comes from the gff file; ^10^ Cell-PLoc 2.0 is used to predict subcellular localization [37,38].

## Data Availability

The sequence data used in the study can be found in Table S1. The *AthSPL* sequences were downloaded from PlantTFDB (http://planttfdb.gao-lab.org/, accessed on 19 November 2022), and the *OsSPL* sequences were downloaded from RGAP (http://rice.uga.edu/, accessed on 19 November 2022).

## Supplementary Materials

The following supporting information can be downloaded at: https://www.mdpi.com/article/10.3390/ijms241210039/s1.
