# Supplementary material for: Genome-Wide Identification and Expression Analysis of the SPL Gene Family in Three Orchids
Source: Int J Mol Sci. 2023 Jun 12;24(12):10039. doi: 10.3390/ijms241210039 (PMC10298685; doi:10.3390/ijms241210039)
Supplement: Supplementary file 1 [file ijms-24-10039-s001.zip › Supplementary Figures.pdf]

Supplementary Figures

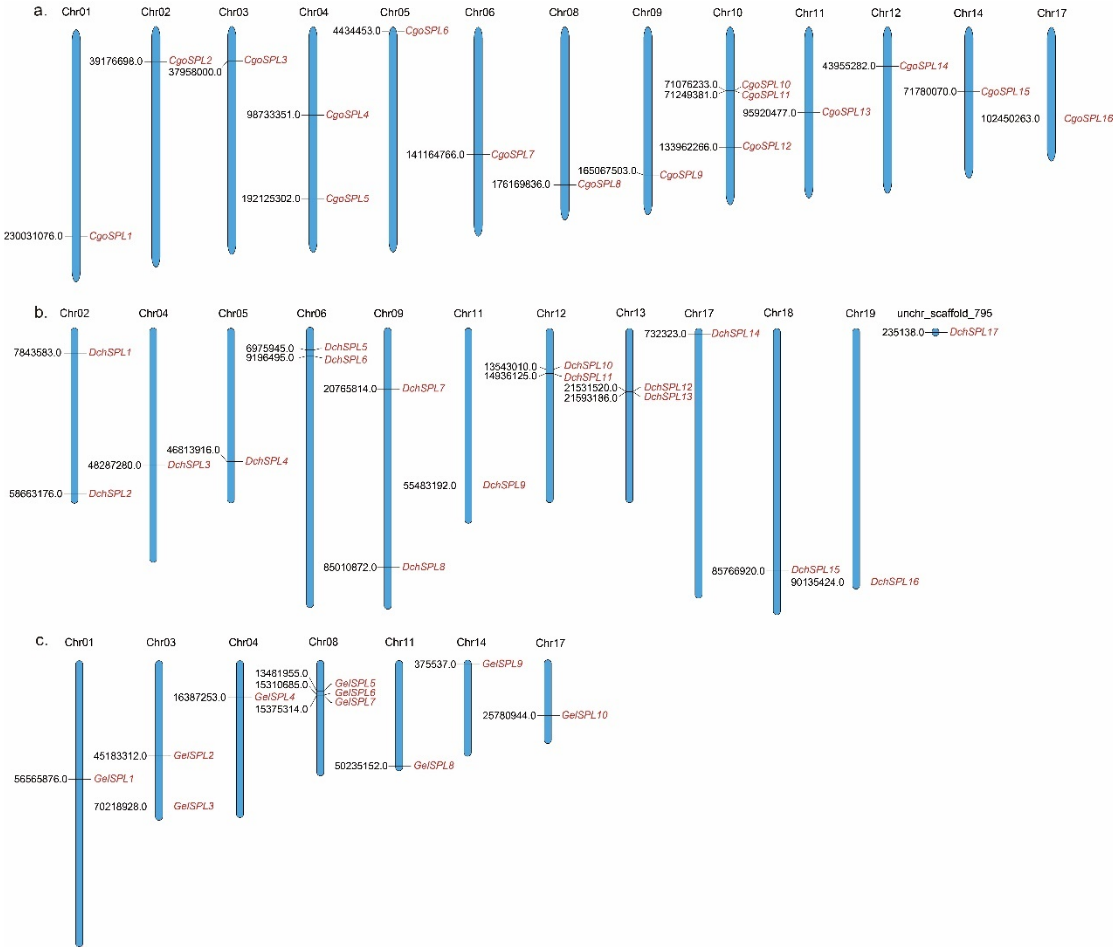

Figure S1. ***SPL*s distribution on chromosomes of *C. goeringii* (a), *D. chrysotoxum* (b), *G. elata* (c).**

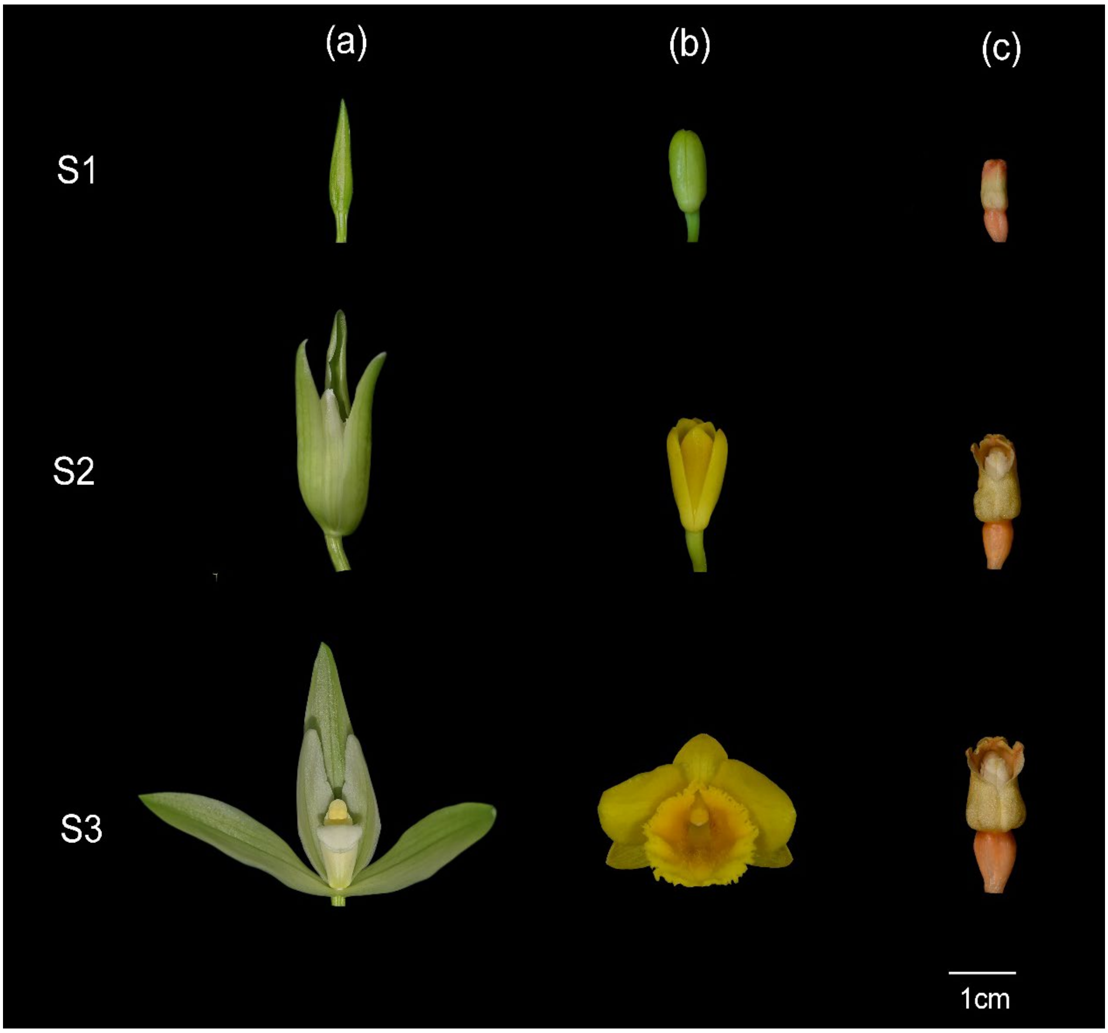

Figure S2. Sampling period of *C. goeringii* (a), *D. chrysotoxum* (b), *G. elata* (c).
